# *YTHDF3*as a prognostic predictive biomarker of thyroid cancer and its correlation with immune infiltration

**DOI:** 10.1186/s12885-023-11361-9

**Published:** 2023-09-19

**Authors:** Yihan Zhang, Ying Chen, Ruihua Chen, Hong Zhou, Yi Lin, Bingxin Li, Huaidong Song, Guoqiang Zhou, Mei Dong, Huanbai Xu

**Affiliations:** 1 Department of Endocrinology and Metabolism, Center for Microbiota and Immunological Diseases, Shanghai General Hospital, School of Medicine, Shanghai Jiao Tong University, Shanghai, China; 2 Department of Endocrinology and Metabolism, Changhai Hospital of Shanghai, Shanghai, China; 3 Department of Molecular Diagnostics & Endocrinology, The Core Laboratory in Medical Center of Clinical Research, Shanghai Ninth People’s Hospital, Shanghai Jiao Tong University School of Medicine, Shanghai, China; 4 Department of General Surgery, The Affiliated Changshu Hospital of Xuzhou Medical University, Changshu, China

**Keywords:** m^6^A RNA methylation regulators, *YTHDF3*, Thyroid cancer, Biomarkers, Immune infiltration

## Abstract

**Purpose:**

Thyroid cancer (TC) is one of the most common endocrine malignancies, and its morbidity continues to rise. N^6^-methyladenosine (m^6^A) RNA methylation, an epigenetic modification, is an important regulator of gene expression in TC. Therefore, it’s worth finding the characteristics and predictive value of the m^6^A RNA methylation regulators in thyroid cancer (TC).

**Method:**

RNA-seq data of TC was downloaded from the Cancer Genome Atlas (TCGA) database to screen out the differential expressed regulators. The absolute contraction selection operator (Lasso) Cox regression was used to construct the risk model of m^6^A methylation regulators. The predictive value of the risk scoring model was evaluated by Kaplan Meier (K-M) analysis and receiver operating characteristic (ROC) curves. The underlying mechanism of m^6^A methylation regulators in TC was predicted by gene set enrichment analysis (GSEA). Further validation was performed by using immunohistochemistry (IHC) and q-PCR. The correlation between risk-related gene and immune infiltration was evaluated by Tumour Immune Estimation Resource (TIMER).

**Results:**

*IGF2BP2, YTHDF1* and *YTHDF3* were screened out as strong independent prognostic factors of TC. Then a risk score model was established to further screen the predictors. Finally, according to the results of overall survival (OS) and clinical characteristics of TC, *YTHDF3* was screened out as a potential predictor. Meanwhile, IHC and qPCR confirmed that *YTHDF3* was expressed differential in TC. The expression of *YTHDF3* was positively associated with the infiltration level of CD4^+^ T cells and macrophages. It was strongly correlated with a variety of immune markers in TC.

**Conclusion:**

We confirmed that *YTHDF3* can be used as a potential prognostic biomarker of TC. It not only plays a decisive role in the initiation and development of TC, but also provides a new perspective for understanding the modification of m^6^A RNA in TC.

## Introduction

Thyroid cancer (TC) is one of the most common endocrine malignancies, and its morbidity continuing to increase [1, 2]. Malignant tumors origin from thyroid follicular epithelial cells, which account for more than 95% in TC, including papillary thyroid cancer (PTC), anaplastic thyroid cancer/undifferentiated thyroid cancer (ATC/UTC) and follicular thyroid cancer (FTC). Medullary thyroid cancer (MTC) origins from paraflular cells of the thyroid gland, with high-grade malignancy [3]. The treatment of TC mainly includes surgical treatment and radioactiveiodine-131 treatment, accompanied by thyroid hormone suppression treatment. In general, the overall prognosis of TC is relatively good, among which the 10-year survival rate of PTC-postoperative patients is more than 90%. However, the problems of rapidly-increasing incidence rate and higher lymph node metastasis rate remain unresolved [4]. The appropriate prognostic factors for TC are still elusive. Therefore, novel molecular biomarkers or prognostic models are urgently needed for early screening of TC. N^6^-methyladenosine (m^6^A) RNA methylation, an epigenetic modification, is emerging as an important regulator of gene expression that affects different biological processes. The changes of m^6^A RNA methylation regulators are associated with cancer [5, 6]. So, m^6^A regulators could be a potential biomarker and provide a new direction of molecular target in TC.

Although recent studies have revealed the epigenetic regulatory function of m^6^A regulatory factor in the immune environment [7], the potential functions and mechanisms of m^6^A RNA methylation regulators in tumor proliferation and tumor immunity remain unclear. In our study, we aim to investigate the correlation between m^6^A RNA methylation regulators with prognosis in thyroid cancer. TC, and screened out tumor-infiltrating immune cells in the TC tumor microenvironment by using tumor immunity estimation resources (TIMER), providing a new idea for understanding the role of m^6^A RNA methylation regulators in anti-tumor immunity.

## Materials and methods

### Selection of m^6^A RNA methylation regulators

The latest research by Li Y.et al. [8] systematically analyzed the molecular alterations and clinical relevance of m^6^A regulators. In their study, 20 m^6^A RNA methylation regulators were screened out with more genetic possibilities, more cancer pathways, and better clinical relevance, which means they deserve further study. Therefore, these 20 m^6^A RNA methylation regulators, including *IGF2BP1, RBM15, FTO, ZC3H13, KIAA1429, YTDHF3, YTHDC2, METTL14, YTHDC1, IGF2BP3, YTHDF1, ALKBH5, IGF2BP2, RBM15B, YTHDF2, HNRN2PB1, METTL3, HNRNPC, RBMX* and *WTAP*, were included in our study for further analysis [6, 8].

### Analyse differential expression levels of regulators

RNA-seq data with clinical information of TC patients was downloaded from the TCGA database (https://tcga-data.nci.nih.gov/tcga/). Specifically, the mRNA expression profile of TCGA-THCA (the Cancer Genome Atlas Thyroid Cancer) was excavated for further study. Then, 496 TC tissues and 58 normal tissues were included in our research for further analysis, after excluding 14 TC tissues with unclear clinical characteristic information. None of the TC patients had been treated. The different expression levels of m^6^A methylation regulators between TC and normal tissue were analyzed by “limma” R package. (log 2-fold change (FC) absolute value > 1 and the adjusted *p* value < 0.05). The “Vioplot” R package was used to compare the differences of m^6^A methylation regulators expression. Search Tool for the Retrieval of Interacting Gene (STRING) database (https://string-db.org/cgi/input.pl) was applied to construct the PPI network of 20 m^6^A RNA methylation regulators, with the score of interactive relationships greater than 0.4. Then, Cytoscape software was used to visualize the PPI network. The correlation coefficient is also calculated by the “corrplot” R package.

### Construct m^6^A-related gene characteristics risk model

Cluster analysis was performed on m^6^A RNA methylation regulators using “Consensus ClusterPlus” R package. Furthermore, TC patients were divided into two subgroups (named Cluster1 and 2, respectively). And then principal component analysis (PCA) and chi-square test were performed to calculate the significance between Cluster1 and Cluster2. Lasso Cox model was established by “survival” R package and “glmnet” R package. The formula of the individual risk score is as follows: Risk Score = ∑coefficient (GENEi) × expression (GENEi). Here, GENEi presented the candidate gene. As a result, patients were classified into low-risk group and high-risk group, according to the median risk scores [9].

### Evaluate the predictive value of methylation regulators

Univariate and multivariate Cox analyses were used to analyse the prognostic value of m^6^A RNA methylation regulators. The independent risk factors include risk scores and various clinical characteristics. Additionally, Multi-ROC curve was used to evaluate the specificity and sensitivity of multiple clinical indicators. Then, Kaplan-Meier curve with Log-rank test was applied to present the overall survival among high-risk and low-risk groups. The ‘survival’ R package was subjected to survival analysis.

### Gene set Enrichment Analysis (GSEA) analysis

To identify potential enriched pathways associated with the meaningful m^6^A RNA methylation regulators, gene set enrichment analysis (GSEA) was performed. And GSEA 3.0 was used for GSEA analysis. H gene sets (h.all.v6.0.symbol.gmt) was selected as hallmark gene set. *p* < 0.05 and FDR < 0.25 was significant.

### TIMER database analysis

TIMER is an interactive Web tool that provides comprehensive and flexible analysis of tumor-infiltrating immune cells, using deconvolution to infer the infiltration abundance of those in different cancer [10]. We analyzed the correlation between *YTHDF3* and the abundance of immune infiltrates. Specifically, the immune infiltration data of B cells, CD4^+^ T cells, CD8^+^ T cells, neutrophils, macrophages, and dendritic cells was used in the analyses. These genetic markers have been cited in previous studies [11–13]. Pearson correlation was used to calculate the relationship between risk scores and immune infiltration.

### Cell culture

The human PTC cell line K1 (catalogue number: 92,030,501), human FTC cell line FTC-238 (catalogue number: 94,060,902), human ATC cell line 8305 C (catalogue number: 94,090,184), human MTC cells TT (catalogue number: 92,050,721) and normal human thyroid follicular epithelial cell line Nthy-ori3-1 (catalogue number: 90,011,609) were purchased from the European Collection of Animal Cell Cultures (ECACC). Dr. Robert Gagel (MD, Anderson Cancer Center, University of Texas) presented the PTC cell line W3.

K1 cells were cultured in DMEM-Ham’s F12-MCDB 105 (2:1:1) (Invitrogen) with 10% fetal bovine serum (FBS) (Gibco), 100 µg/mL streptomycin, and 100 U/mL penicillin. W3 cells were cultured in DMEM with 10% FBS, 100 µg/mL streptomycin and 100 U/mL penicillin. FTC-238 cells were cultured in DMEM-Ham’s F12(1:1) containing 10% FBS, 2mM glutamine, 100 U/mL penicillin and 100 mg/mL streptomycin. 8305 C cells were cultured in EMEM(HBSS) (Thermo Fisher Scientific) with 10% FBS, 1% nonessential amino acids (NEAA), 2mM glutamine, 100 µg/mL streptomycin and 100 U/mL penicillin. TT cells were cultured in Ham’s F12 with 10% FBS, 2mM glutamine, 1%NEAA,1% sodium pyruvate (NaP), 100 U/mL penicillin and 100 mg/mL streptomycin. Nthy-ori3-1 cells were cultured in RPMI-1640 (Invitrogen) containing 10% FBS, 100 U/mL penicillin, and 100 mg/mL streptomycin. The cells were cultured in incubators containing 5% CO2 at 37 °C.

### RNA extraction and qPCR and immunohistochemistry (IHC)

Total RNA was isolated from cells using the TRIzol (Life Technologies), and reverse transcribed. Followed by qPCR with Power SYBR Green PCR Master Mix (Eppendorf), each gene’s relative expression levels were calculated and normalized to β-actin as an endogenous control using the 2-^△△^CT method. Takara kit used for cDNA synthesis. Each sample was repeated at least three times. The forward primers sequences of *YTHDF3* is 5’-CTGGAACAATCACTCGGCCA-3’, the reserve sequences is 5’-CCTTGCCCTTTAGGTCTCTGA-3’. The forward sequence of β-actin is 5’-CACCTTCTACAATGAGCTGCGTGTG-3’, the reserve sequences is 5’-ATAGCACAGCCTGGATAGCAACGTAC-3’. The primers (random hexamers) were synthesized by Shanghai Sangon (Shanghai, China).

Thirty pairs of TC (12 PTC, 6 FTC, 6 ATC, 6 MTC) and adjacent normal thyroid paraffin-embedded tissue were collected from the Shanghai General Hospital during the period of September 2017 to May 2020. The experiments were approved by the Ethical Committee of Shanghai General Hospital. All patients have signed written informed consent. All samples were incubated using rabbit monolyclonal anti-*YTHDF3* antibody (1:100 dilution; Cat. ab220161; Abcam; USA) overnight at 4 °C. Rabbit Anti-Mouse IgG H&L (HRP; ab6728; Abcam; USA) was used as secondary antibody. The standard procedures of IHC were described in detail previously [14]. We scanned the slices by Pannoramic (3DHISTECH) and ran caseviewer (2.4.0.119028) to take pictures. In the quantization process, four classes (from 1 to 4) were assigned based on the estimated positive area, which corresponding positive area is 0%, < 10%, 10–50%, > 50%, respectively.

### Statistical analysis

All statistical analysis was applied by R version 3.6.1. All data is presented as the mean ± standard deviation (SD). Two-sided Student’s T tests were used in significance test of two group comparisons. *p* < 0.05 was considered to represent a statistical significance.

## Results

### Different expression of 20 m^6^A RNA methylation regulators in TC

**Fig. 1:**
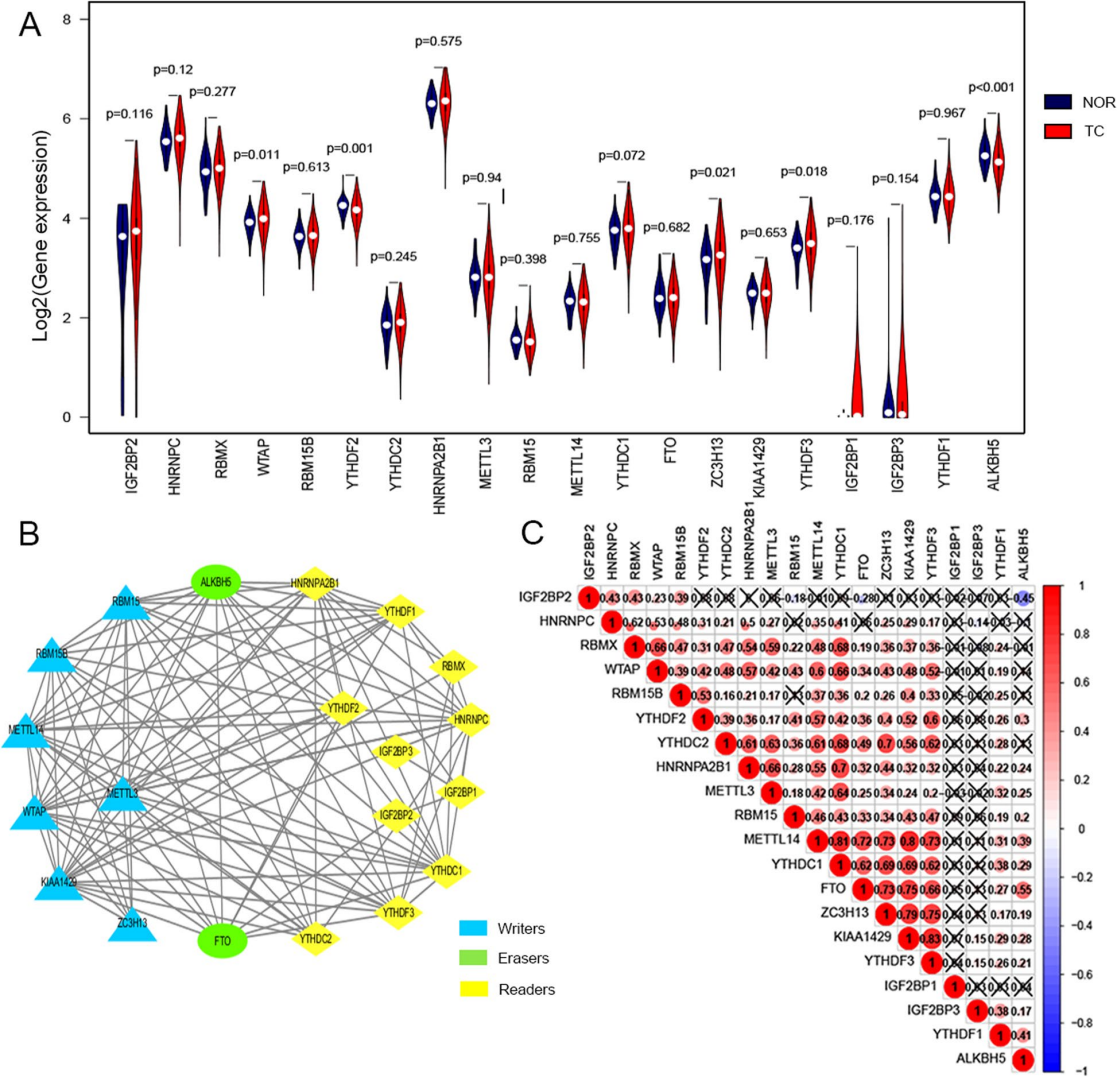
Differential expression and correlation of m^6^A RNA methylation regulators in TC. **A** The Vioplot of the expression profiles of 20 m^6^A RNA methylation regulators in normal thyroid and TC (red represents TC tissues, blue represents normal tissues). **B** The PPI network of the 20 m^6^A methylation regulators constructed using STRING. **C** Spearman correlation analysis of the 20 m^6^A methylation regulators. “X” indicates significant uncorrelation

We analyzed different expression of 20 m^6^A RNA methylation regulators between TC tissues and normal tissue. As a result, *ALKBH5* and *YTHDF2* were down-expressed in TC tissue, while *WTAP, YTHDF3* and *ZC3H13* were up-expressed in TC tissue (*P* < 0.05) (Fig. 1A).

In order to infer the relationship between these 20 m^6^A RNA methylation regulators, the PPI network was constructed based on the STRING database (Fig. 1B) and their correlation was calculated by “corrplot” R package (Fig. 1C). The line connecting each m^6^A RNA methylation regulators means the association between two methylation factors. The more lines there are, the stronger the relationship between the two factors. Figure 1B showed that *WTAP, ZC3H13* present the function of writer, and *YTHDF3* presents the function of readers. In addition, *METTL14, YTHDC1, FTO, ZC3H13, KIAA1429* and *YTHDF3* were significantly correlated with each other in TC. Among them, the expression of *YTHDF3* was significantly correlated with *KIAA1429* (*r* = 0.83), *YTHDC2* (*r* = 0.62) and *YTHDF2* (*r* = 0.60) (Fig. 1C). What’s more, the expression of *HNRNPA2B1* was significantly associated with *YTHDC1* (*r* = 0.7) (Fig. 1C). These may suggest that *YTHDF3* may be a methylation factor worth further studying.

### Identify risk characteristics of three m^6^A RNA methylation regulators

**Fig. 2:**
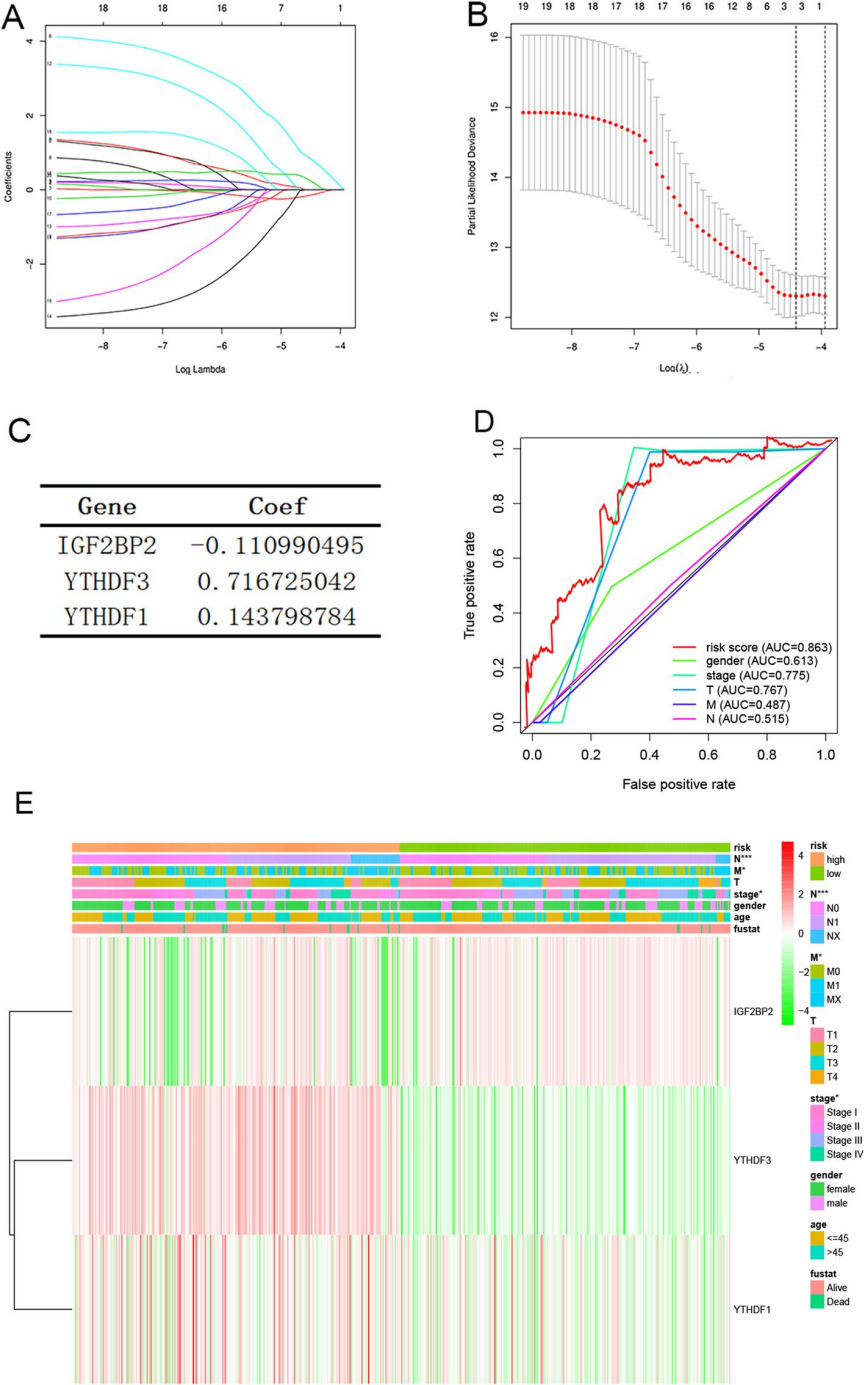
Identification of risk characteristics of m6A RNA methylation regulators associated with prognosis. **A** LASSO coefficient profiles of 20 m^6^A RNA methylation regulators. **B** Log (lambda) sequence was used to generate a coefficient profile plot. **C **The coefficient of three prognosis-related genes. **D** Multi-ROC curve analyses of the predictive value of clinical indicators in TC patients. **E** The heatmap shows risk scores, clinical phenotypes, and gene expression (*IGF2BP2, YTHDF1*, and *YTHDF3*) in patients with TC. Chi-square test was used to assess the relationship between risk score and clinical phenotype (*p < 0.05, **p < 0.01, ***p < 0.001)

 To understand the risk characteristics of 20 m^6^A RNA methylation regulators in TC, LASSO regression analysis was used to find potential risk-related genes and assess their independent prognostic value. LASSO coefficient profiles of 20 m^6^A RNA methylation regulators were listed in Fig. 2A. And a coefficient profile plot generated against the log (lambda) sequence was listed in Fig. 2B. The coefficient of three risk -related genes were listed in Fig. 2C. As a result, *IGF2BP2, YTHDF1, YTHDF3* were screened out as three candidate genes. According to the regression coefficient of these three genes, an individual risk rating model was set up, in order to assess and predict the risk of each TC patient. The formula is as follows: Risk score = (-0.111 × *IGF2BP2*) + (0.144 × *YTHDF1*) + (0.717 × *YTHDF3*). Then, in order to test the prognostic value of these three genes, TC tissues were divided into low-risk subgroup and high-risk subgroup according to the median risk score. Furthermore, Multi-ROC curve was used to evaluate the specificity and sensitivity of multiple clinical indicators, shown in Fig. 2D. The AUC of risk score was 0.863, which means our model had the better predictive power than other indicators, such as: gender (AUC = 0.613), pathological stage (AUC = 0.775), tumor size (T) (AUC = 0.767). In order to compare the clinical characteristics with TC, the patients with TC were divided into two groups: high risk group and low risk group. Then they mapped the relationship between risk scores (high- and low risk groups) and clinicopathological characteristics (including tumor site, metastasis, age, and sex). Understandably, most patients with TC from high-risk group had an increased expression of *YTHDF3* and *YTHDF1*, and significant association was established with the regional lymph node(N), metastasis(M) and pathological stage (Table 1; Fig. 2E).

**Table 1:** The Clinicopathological Information of The TC Patient

Total patients (496)			High-risk group (247)		Low-risk group (249)	
	Number	Percentage (%)	Number	Percentage (%)	Number	Percentage (%)
**Age**						
≤ 45	235	47%	125	51%	110	44%
> 45	261	53%	122	49%	139	56%
**Gender**						
Female	362	73%	184	74%	178	71%
Male	134	27%	63	26%	71	29%
**Stage**						
I	278	56%	147	60%	131	53%
II	52	10%	26	11%	26	10%
III	111	22%	47	19%	64	26%
IV	55	11%	27	11%	28	11%
**Stage T**						
T1	139	28%	69	28%	70	28%
T2	164	33%	88	36%	76	31%
T3	170	34%	81	33%	89	36%
T4	23	5%	9	4%	14	6%
**Stage M**						
M0	279	56%	130	53%	149	60%
M1	9	2%	6	2%	3	1%
Mx	208	42%	111	45%	97	39%
**Stage N**						
N0	227	46%	105	43%	122	49%
N1	221	45%	111	45%	110	44%
Nx	48	10%	31	13%	17	7%

### Risk model evaluated and predicted the poor prognosis of TC

**Fig. 3:**
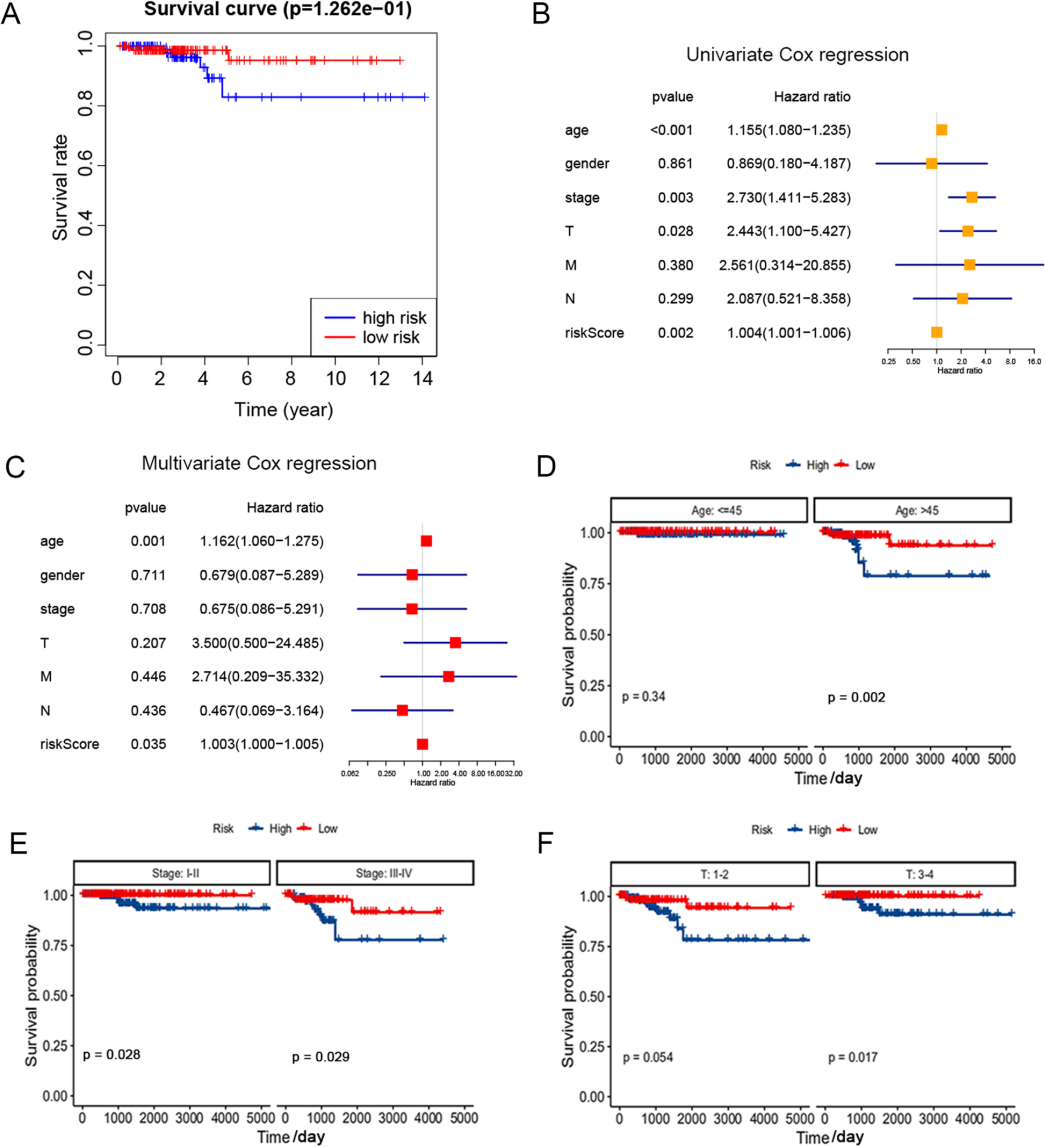
Analysis of prognostic factors for TC patients. **A** Kaplan-meier curve analysis between high-risk and low-risk TC patients. **B** A univariate Cox regression model of the relationship between TC clinicopathological factors and prognosis. **C** A multivariate Cox regression model of the relationship between TC clinicopathological factors and prognosis. **D** Age - stratified subgroup analysis was performed for overall survival between high - and low-risk groups. **E** Stage - stratified subgroup analysis was performed for overall survival between high - and low-risk groups. **F** T - stratified subgroup analysis was performed for overall survival between high - and low-risk groups

Then, the results of survival curve showed that the overall survival (OS) of the high-risk group decreased significantly, compared with the low-risk group (*p* < 0.001) (Fig. 3A). Next, univariate and multivariate COX regression analysis was conducted to compare clinicopathological characteristics (including age, gender, stage, tumor size (T), node (N), and metastasis (M)) in the predictive model, in order to find out whether these risk characteristics could be independent factors for TC. As a result, the age at diagnosis (*p* < 0.001), pathological stage (*p* = 0.003), stage T (*p* = 0.028) and risk score (*p* = 0.002) were correlated with OS in univariate Cox regression analysis (Fig. 3B), while only age (*p* = 0.001) and risk score (*p* = 0.035) remained significantly correlated with OS in multivariate Cox regression (Fig. 3C). Then, based on the results of univariate analysis, a subgroup analysis was conducted on the clinicopathological characteristics with significant significance in age, gender and pathological stage, so as to evaluate the predictive prognostic value of risk characteristics of TC patients. As shown in Fig. 3D-F, compared with the low-risk group, the OS of high-risk patients in each subgroup was significantly lower (*p* < 0.05), suggesting that the high-risk group related to the older, later stage, and higher T classification level has poor prognosis in TC. These results indicate that the risk model based on the expression of m^6^A RNA methylation regulators can evaluate and predict the prognosis of TC.

### *YTHDF3* was selected as hub regulator and its potential related pathways

**Fig. 4:**
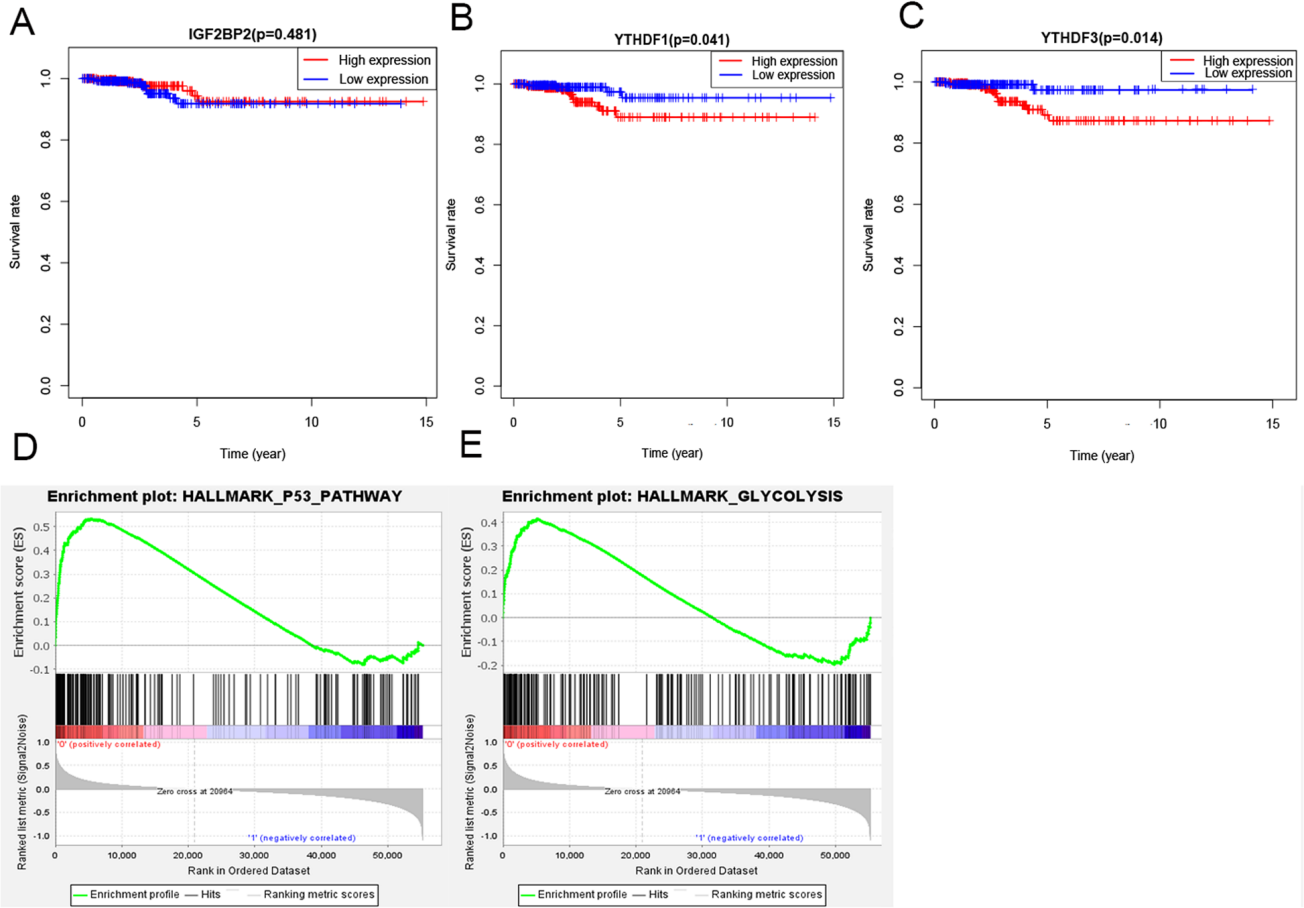
Prognosis value and underlying mechanism of prognostic genes. **A** Subgroup analysis of overall survival between the *IGF2BP2* high expression group and the low expression group. **B** Subgroup analysis of overall survival between the *YTHDF1* high expression group and the low expression group. **C** Subgroup analysis of overall survival between the *YTHDF3* high expression group and the low expression group. **D**, **E** GSEA analysis revealed that the signaling pathways enriched by high expression of *YTHDF3* were the P53 pathway, Glycolysis

In order to find the hub regulator among the risk models, survival analysis among these three regulators was conducted. The results of survival analysis showed that the high expression group of *YTHDF3* (*p* = 0.041) and *YTHDF1* (*p* = 0.014) has lower survival rate (Fig. 4B, C). However, there was no significant difference between high and low expression group of *IGF2BP2* (Fig. 4A). Combine the results of Fig. 1A, the analysis of mRNA expression showed that *YTHDF1* was not significantly expressed among TC (*p* > 0.05). So, *YTHDF3* was selected as hub gene and then included in Gene Set Enrichment Analysis (GSEA) for pathway analysis. Then, GSEA showed that the signaling pathways with high *YTHDF3* expression were P53 pathway (NES = 1.94, NOM *p* = 0.006, FDR q = 0.068), Glycolysis (NES = 1.74, NOM *p* = 0.009, FDR q = 0.211) (Table 2; Fig. 4D, E). In a word, the prognostic value and potential mechanism of *YTHDF3* were indicated.

**Table 2:** GSEA analyzes the regulatory pathways or biological functions related to YTHDF3 gene expression

Gene Expression	Hallmark Gene Sets	NES	NOM p-val	FDR q-val
High	P53_PATHWAY	1.94	0.006	0.068
	GLYCOLYSIS	1.74	0.009	0.211

*NES *Normalized enrichment score. NOM *p*-val stands for *P* value, representing the credibility of the enrichment results; FDR q-val 'stands for Q value, which is the *P* value corrected after multiple hypothesis testing. GSEA USES *P* value < 5% and Q value < 25% to filter the results

### *YTHDF3* correlated to immune infiltration

**Fig. 5:**
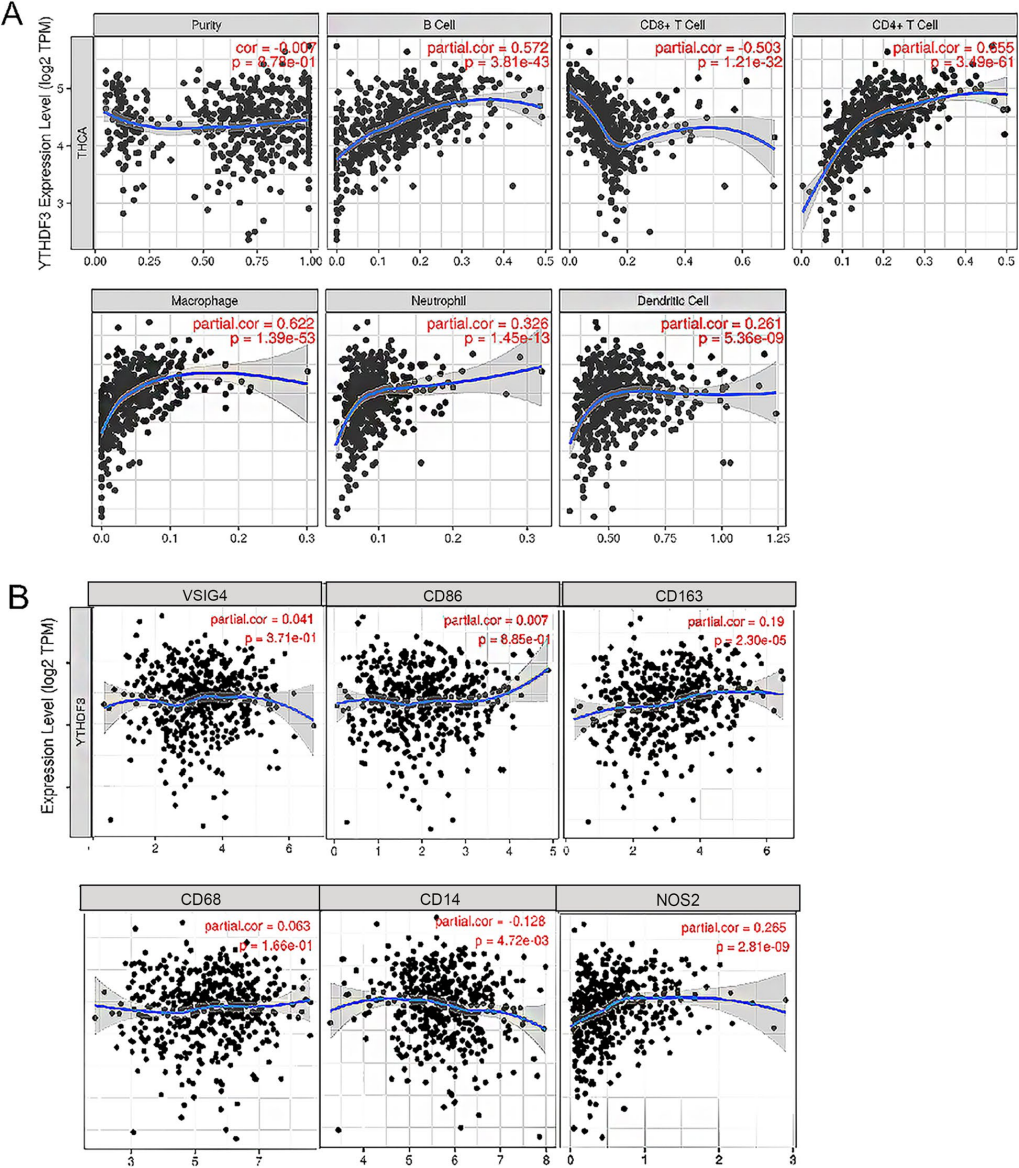
Correlation between *YTHDF3* and immune infiltration in TC. **A** tumor-infiltrating immune cells was investigated through related modules (B cells, CD4^+^ T cells, CD8^+^ T cells, neutrophils, macrophages, and dendritic cells). *r* > 0.05 indicates strong correlation, *r* < 0.05 indicates weak correlation. **B** The X axis represents the associated immune markers, and the Y axis represents *YTHDF3*. Log2RSEM was used to display gene expression. Markers include CD14, CD86 of monocytes; CD68 of TAMs (tumor-associated macrophages); NOS2 of M1 macrophages; and VSIG4, CD163 of M2 macrophages. *r* > 0.05 indicates strong correlation, *r* < 0.05 indicates weak correlation

In recent years, immune infiltration has investigated potential molecular characterization of tumor-immune interactions, and plays a more and more important role in cancer therapy. So, it’s necessary to analyse the immune infiltration among TC. The expression of *YTHDF3* has significant positive correlations with infiltrating levels of B cells (*r* = 0.572, *p* = 3.81e-43), CD8^+^ T cells (*r* = 0.503, *p* = 1.21e-32), CD4^+^ T cells (*r* = 0.655, *p* = 3.49e-61), macrophages (*r* = 0.622, *p* = 1.39e-53), neutrophils (*r* = 0.326, *p* = 1.45e-13) and DCs (*r* = 0.261, *p* = 5.36e-09) in Fig. 5A. Moreover, we found that CD14 of monocytes (*r* = -0.128, *p* = 4.72e-03), NOS2 of M1 phenotype (*r* = 0.265, *p* = 2.81e-09), CD163 of M2 phenotype (*r* = 0.19, *p* = 2.30e-05) are significantly correlated with *YTHDF3* expression in TC (*p* < 0.001) in Fig. 5B.

### *YTHDF3* was expressed in cells and tissues

**Fig. 6:**
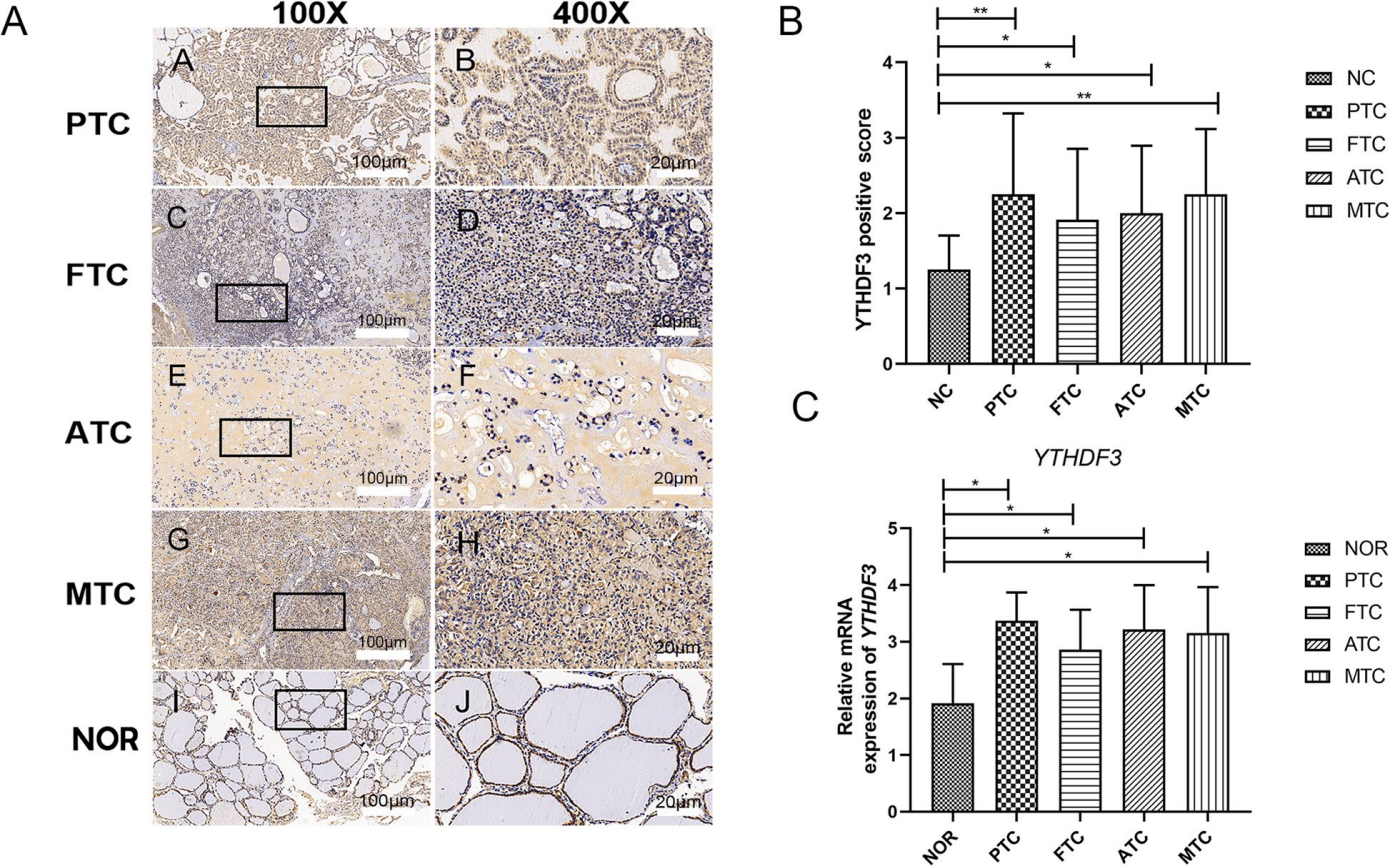
Immunohistochemistry and q-PCR verified the expression level of  *YTHDF3* between TC and normal tissues. **A** Immunohistochemical expression of *YTHDF3* on TC (PTC, FTC, ATC, MTC) tissues. The brown area in Fig. 6A marked the positive staining of *YTHDF3* in tumor tissues. The scale bar is located in the lower right corner of each group of images, with a scale of 100 μm under 100x microscope and 20 μm under 400x microscope. **B** The quantification of immunohistochemistry based on positive area score. **C** The expression levels of *YTHDF3* mRNA in human PTC, FTC, ATC and MTC cell and the normal thyroid follicular epithelial cell were analyzed by q-PCR. The data represents the mean ± S.E.M. (* *p <* 0.05, **p < 0.01, ***p < 0.001). The human PTC cell line K1, human FTC cell line FTC-238, human ATC cell line 8305 C, human MTC cells TT and normal human thyroid follicular epithelial cell line Nthy-ori3-1 were used in q-PCR. (* *p <* 0.05, **p < 0.01, ***p < 0.001)

To further verify our analysis results, immunostaining of *YTHDF3* in TC (including papillary thyroid carcinoma (PTC), follicular thyroid carcinoma (FTC), medullary thyroid carcinoma (MTC), and anaplastic thyroid carcinoma (ATC)) and normal thyroid tissues were displayed in Fig. 6A, which proved that compared with normal tissues, *YTHDF3* was up-regulated in TC. And immunohistochemistry can also be quantified in Fig. 6B. And then, the human PTC cell line K1, human FTC cell line FTC-238, human ATC cell line 8305 C, human MTC cells TT and normal human thyroid follicular epithelial cell line Nthy-ori3-1 were used in q-PCR. As a result, compared with normal thyroid tissue, the mRNA expression of *YTHDF3* in PTC, FTC, ATC, and MTC was up-regulated by q-PCR (Fig. 6C), which is basically consistent with TCGA dataset analysis. Through these two validations, *YTHDF3* was validated as a predictive biomarker.

## Discussion

TC is the most common endocrine cancer, with a rapidly increasing incidence rate of cancer worldwide [15]. Recent studies have shown that epigenetics, such as methylation regulators and gene mutation, play a key role in the initiation and progression of TC [16, 17]. More and more evidence indicates that m^6^A RNA methylation regulators play a role as RNA transcriptional modification in tumor [5, 18, 19].

At present, some research has explored the potential characteristics of m^6^A RNA methylation regulators in the prognosis of TC. Xu, N.et al. [20] have demonstrated 4 m^6^A methylation regulators related to the prognosis of DTCs, named *HNRNPC, WTAP, ALKBH5*, and *ZC3H13*. Compared with their study, the samples we selected in our study have deleted samples with unclear clinical characteristic information. Therefore, the results of our study were different from their studies. At the same time, it means our results are more accurate. Meanwhile, Wang, X.et al. [14] have shown that *YTHDF3* is related to OS of PTC through bioinformatics analysis, which is consistent with our conclusion. More importantly, our study was the first one to verify *YTHDF3* in experiment. Specifically, we verified the expression level of *YTHDF3* in TC by IHC and q-PCR, which means the results were validated at both protein and RNA levels. And we are the first one to clarify that *YTHDF3* is associated with poor prognosis in TC. Therefore, *YTHDF3* can be a potential prognostic biomarker and provide a new direction of molecular target in TC.

Our study was the first to conduct GSEA analysis according to the expression level of *YTHDF3*. The results of GSEA showed that high expression of *YTHDF3* was associated with p53 pathway, which has never been reported before. P53 pathway is the most reported signaling pathway in human cancer, which influences the procession of cancer through gene mutation, cell cycle progression, DNA damage and so on [21]. Previous studies have reported that *YTHDF3* involves the activation of several pathways, such as protein secretion, androgen response and the TGF-β signaling pathway [8].

The results of immune infiltration strongly suggest that *YTHDF3* may play a role in the immune infiltration of TC, especially the immune infiltration of CD4^+^T cells and macrophages. And it may affect the recruitment of TC immune infiltrating cells and the regulation of tumor-associated macrophages (TAM) polarization. Previous study showed that knockout of *YTHDF3* in human CD4^+^ T-cells increases infection supporting the role of *YTHDF3* as a restriction factor [22]. Another study shows that N1-Methyladenosine (m1A) regulation associated with the pathogenesis of abdominal aortic aneurysm through *YTHDF3* modulating macrophage polarization [23]. And it’s worth noting that we showed the potential heterogeneity of *YTHDF3* in different subtypes of TC. The immune microenvironment between subtypes of TC is different, so the clinicopathologic correlations and prognostic impact of *YTHDF3* in TC are histotype-dependent.

Some studies showed that a triple-negative breast cancer (TNBC) cell line expressing p53(R280K), when exposed to TNF, secretes chemokines that modulate recruitment of immune cells to the tumor. It’s suggested that mutp53 may shape the tumor immune infiltrate [24]. What’s more, lots of paper show the relationship of glycolysis and immune [25]. There’s a study shows that enhanced glycolysis activity of breast cancer was associated with pro-tumor immunity. The interaction between tumor glycolysis and immune/inflammation function may be mediated through IL-17 signaling pathway [26]. And another paper reveled the relationship of p53 pathway and CD4^+^ T cell: Human umbilical cord-derived mesenchymal stem cell therapy ameliorates lupus through increasing CD4^+^ T cell senescence via MiR-199a-5p/Sirt1/p53 axis [27]. So, we suspect that these two signaling pathways are involved in tumor immunity and thus influence the progression of TC.

There is still some room in our research could be improved: multi-omics could be applied in our study to investigate associations among genomics, transcriptomics, proteomics, epigenomics and so on. Multi-omics analysis of thyroid cancer may provide a novel perspective on gene regulation network, which can deeply understand the regulation between diseases and molecule [28]. What’s more, it offers a new strategy for establishing the relationship between immune micro-environment and tumor proliferation and migration [29].

In conclusion, we assume that the promotion of *YTHDF3* m^6^A methylation regulator affects gene silence and alternative splicing patterns, activating p53 pathway and glycolysis pathway, affecting CD4 + T cells and macrophages, causing the occurrence and progression of thyroid cancer. Therefore, *YTHDF3* could be a potential biomarker of poor prognostic and provide a new direction of molecular target. However, the specific mechanism of *YTHDF3* in TC and its epigenetic regulation in immune environment still need to be further explored.

## Data Availability

The data that support the findings of this study are openly available on TCGA website. The link to the data is following: https://tcga-data.nci.nih.gov/tcga/.
